# Single-molecule and super-resolution imaging of transcription in living bacteria

**DOI:** 10.1016/j.ymeth.2017.04.001

**Published:** 2017-05-01

**Authors:** Mathew Stracy, Achillefs N. Kapanidis

**Affiliations:** Biological Physics Research Group, Clarendon Laboratory, Department of Physics, University of Oxford, Oxford OX1 3PU, United Kingdom

**Keywords:** Super-resolution imaging, Transcription, Quantitative microscopy, PALM, Single-particle tracking, Live cells, FP, fluorescent protein, PAFP, photoactivatable fluorescent protein, RNAP, RNA polymerase, dSTORM, direct stochastic optical reconstruction microscopy, PALM, photoactivated localization microscopy, sptPALM, single-particle tracking PALM, bp, basepair, SIM, structured illumination microscopy, EMCCD, electron multiplying charge coupled device, FRAP, fluorescence recovery after photobleaching, MSD, mean squared displacement, FROS, fluorescent repressor operator system

## Abstract

*In vivo* single-molecule and super-resolution techniques are transforming our ability to study transcription as it takes place in its native environment in living cells. This review will detail the methods for imaging single molecules in cells, and the data-analysis tools which can be used to extract quantitative information on the spatial organization, mobility, and kinetics of the transcription machinery from these experiments. Furthermore, we will highlight studies which have applied these techniques to shed new light on bacterial transcription.

## Highlights

• Super-resolution imaging and tracking of RNA polymerase [RNAP] in living bacteria.
• RNAP tracking reports on its intracellular mobility and spatial distribution.
• RNAP spatial and diffusion profile is sensitive to antibiotics and growth media.
• RNAPs on highly expressed genes are found in clusters at the nucleoid periphery.
• RNAP spends most of its promoter search time bound non-specifically to DNA.

## 1 Introduction

Transcription is one of the most fundamental processes necessary for life, being the first step in gene expression and ultimately responsible for how both eukaryotic and prokaryotic cells respond to changes in their environment. In bacteria, unlike eukaryotes, there is only a single type of RNA polymerase (RNAP) responsible for transcription of both coding and non-coding RNA. RNAP is a multi-subunit protein machine made up of a beta and a beta prime subunit, two alpha subunits, and an omega subunit. In order to bind promoters, the RNAP core associates with transcription initiation sigma factors (σ factors) to form the RNA polymerase holoenzyme; in the case of the *Escherichia coli* housekeeping σ factor (σ^70^), this association forms a 450 kDa holoenzyme [1]. Sigma factors reduce the affinity of RNAP for non-specific DNA while increasing specificity for promoters.

There are ∼2000 σ^70^-specific promoters in *E. coli* [2], each containing a core sequence of ∼40 base pairs (bp) in length, with two short sequences approximately −10 and −35 bp upstream of the transcription start site. Taken together, these promoter sequences account for less than 2% of the *E. coli* genome [3]. In order to locate a promoter, an RNAP molecule must therefore discriminate between vast amounts of nonspecific DNA.

**Fig. 1:**
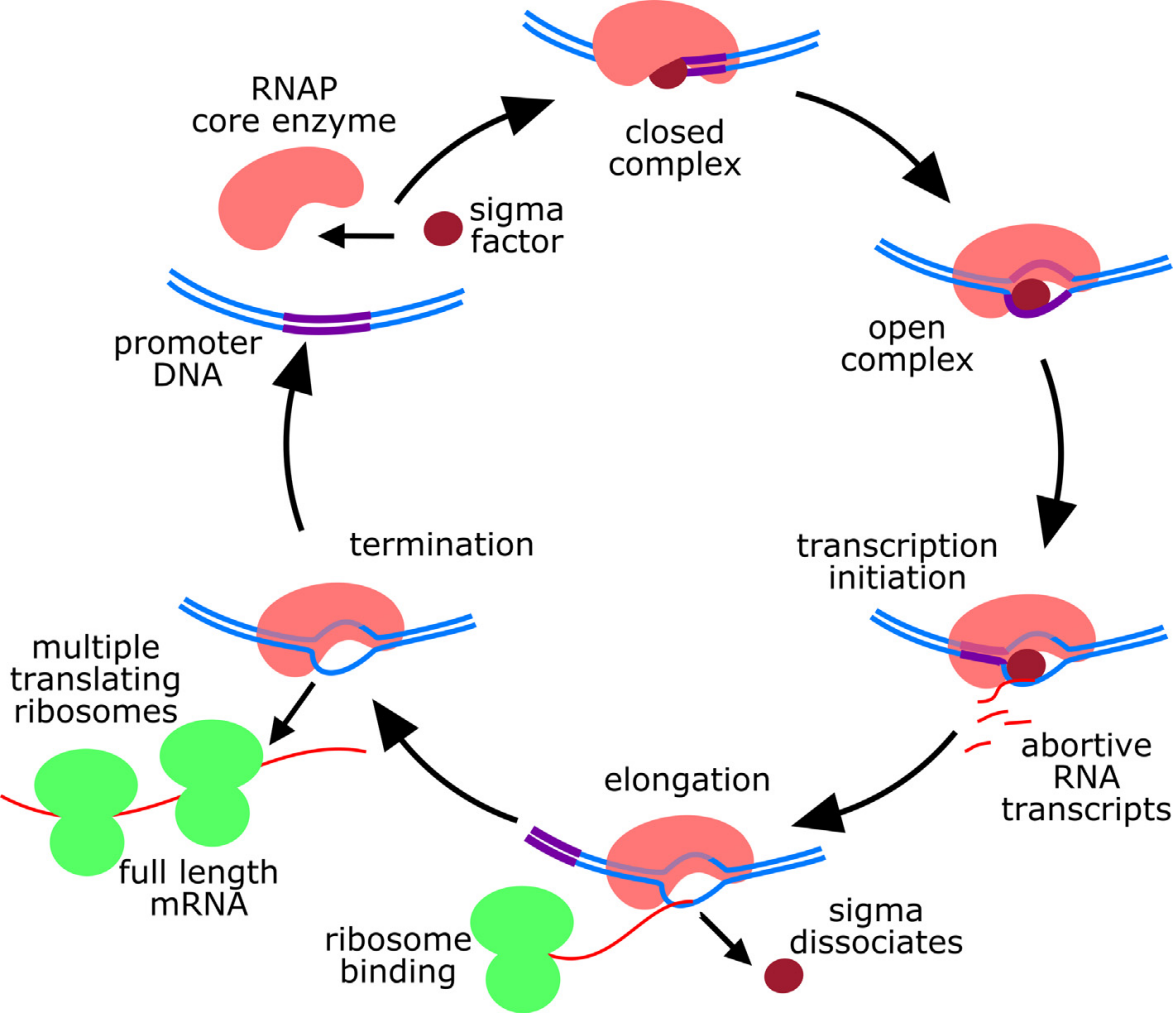
The transcription cycle. RNAP associates with a sigma factor before binding to a promoter site. After initial binding, the enzyme opens a bubble in the duplex DNA to form an ‘open complex’. From here, it can initiate transcription; however, on many promoters, the polymerase makes several attempts to start transcribing, generating short abortive RNAs [14]. Once past the ∼10th nucleotide, the RNAP breaks its interactions with promoter DNA and enters into processive synthesis of RNA as an ‘elongation complex’. At some point during elongation, the sigma factor usually dissociates from the core enzyme [6]. Finally, RNAP reaches the end of the gene, and the RNA transcript and the core enzyme dissociate from DNA.

After initial binding to the promoter, RNAP opens a bubble in the duplex DNA to form an ‘open complex’ and begins transcription (Fig. 1) [4], [5]. In bacteria, transcription and translation are not segregated, and ribosomes can form on the nascent transcript as soon as the ribosome binding site has emerged from the RNA-exit channel of RNAP. At some point during elongation, the sigma factor usually dissociates and is free to associate with another core enzyme [6]. Finally, RNAP reaches the end of the gene, and the RNA transcript and the core enzyme dissociate from DNA.

At the molecular level, much of our understanding of transcription is based on *in vitro* experiments performed using purified proteins and DNA. The finest level of detail has been achieved through X-ray crystallography, allowing the precise interactions between the bases on the DNA and the amino acid residues on the transcription machinery to be determined. However, the ‘snapshots’ from crystallography are poorly suited to studying dynamic behavior. To complement structural information from crystallography, *in vitro* single-molecule experiments are becoming increasingly popular tools to study transcription, since they can determine the kinetics of these interactions by directly observing the behavior of individual molecules [7], [8], [9], [10], [11], [12], [13], [14], [15], [16], [17]. While *in vitro* single-molecule techniques have been used to great effect in elucidating molecular behavior, care must be taken when inferring the physiological relevance, since these experiments are performed on highly simplified systems and in isolation from the rest of the cellular components.

At a larger scale, transcription interactions can be put in the context of the complete chromosome with tools like chromatin immunoprecipitation (ChIP), which uses lysates from a population of cells to determine the specific binding sequences of proteins of interest, and the protein occupancy of genomic sites under different physiological conditions [18]. Furthermore, next generation sequencing allows large scale analysis of the transcriptome, shedding light on the levels of gene expression. However, such techniques cannot report on the spatial organization of transcription in cells, or the kinetics involved, and do not provide information on the heterogeneity between cells since they derive their results from the mean properties of populations of cells.

With *in vivo* single-molecule and super-resolution techniques, transcription machinery can be visualized in living cells [19], [20], [21], [22], [23], [24], shedding new light on the spatial organization, DNA search process and binding kinetics of the proteins involved [25]. Here, we detail methods for performing these experiments, from constructing a single-molecule microscope and imaging samples, to quantitative data analysis. We highlight the advantages and challenges of applying these techniques in living cells. In particular, we focus on photoactivation localization microscopy (PALM), and its combination with single-particle tracking. We further show how these methods have been used to answer key questions about bacterial transcription.

## 2 Experimental methods

### 2.1 Super-resolution fluorescence microscopy

The transcription machinery can be imaged inside living bacteria using fluorescence microscopy. However, while conventional fluorescence microscopy can report on large cellular features, details are lost below the diffraction limit of light (∼200 nm). Over the past decade, several new techniques have been developed to beat the diffraction limit, allowing light microscopy to achieve much higher resolution than ever thought possible. These super-resolution techniques fall broadly into two categories: single-molecule localization methods, where fluorescence signal is collected for each labelled molecule individually, and ensemble imaging methods, where fluorescence from an ensemble of molecules is collected [26]. Each of these techniques has its own advantages, limitations, and caveats.

The ensemble imaging methods that break the diffraction limit rely on illuminating the sample with patterned excitation; these methods include stimulated-emission depletion (STED) microscopy, and structured illumination microscopy (SIM), with the latter being a very popular route to super-resolution. SIM increases resolution by using sinusoidal patterned excitation light [27]; the interference pattern of the sample structure and the excitation pattern contains otherwise-unobservable information about the sample. Multiple images are taken of the same sample but with different angles and phases of the excitation pattern. As the excitation pattern is known, the final image can be computationally reconstructed from the multiple snapshots, allowing a resolution of ∼100 nm.

One of the key advantages of patterned illumination techniques such as SIM, is that they require no special fluorophores or sample preparations, they are hence ‘backwards compatible’ with previously labelled samples and can be readily used in live cells. SIM is also well suited to acquiring multi-color super-resolved images. On the other hand, SIM requires multiple images per field of view and is linked to rapid photobleaching; as a result, SIM is not well suited to samples with low copy numbers of labelled molecules.

**Fig. 2:**
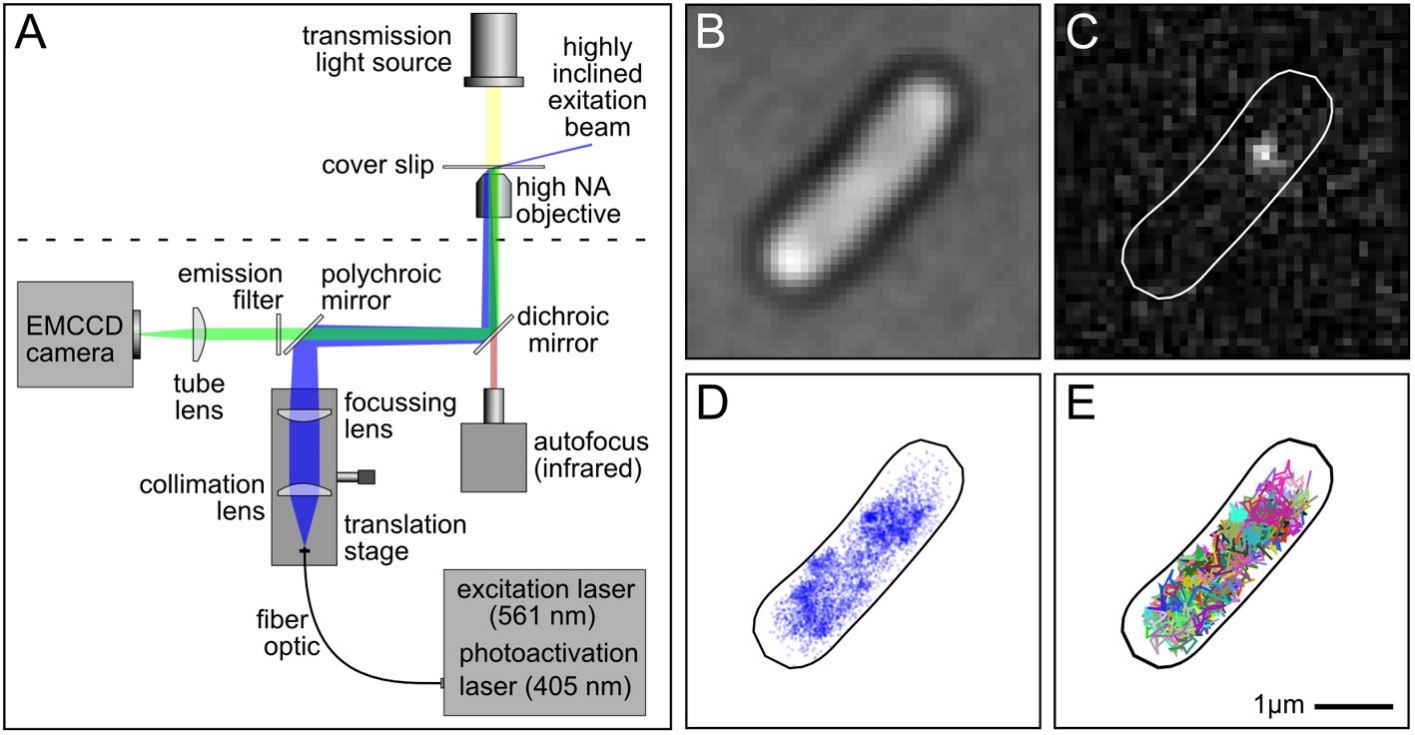
*In vivo* single-molecule fluorescence microscopy. A) Schematic of an example microscope setup for single-molecule microscopy. Photoactivation and excitation lasers are coupled into an optical fiber. Light from the fiber output is collimated and focused on the back focal plane of the objective. Translation of the fiber output, collimation and focusing lenses allows for control of the incident angle of the beam at the coverslip. The emission signal is filtered from the excitation light with a polychroic mirror and focused onto an EMCCD camera. Transmission light is provided by an LED above the sample, and autofocus is provided by an infrared LED and a position-sensitive photo detector. B) An example transmission image of a live *E. coli* cell. C) A single frame of a PALM movie showing the fluorescence image from a single labelled RNA polymerase molecule. D) A super-resolved image of RNAP generated from imaging and localizing all available RNAP molecules over 20,000 frames. E) Trajectories of RNAP; each color corresponds to a single track. (For interpretation of the references to color in this figure legend, the reader is referred to the web version of this article.)

In contrast to the ensemble super-resolution methods, localization-based methods have arisen in a large part due to breakthroughs in the fluorophores used to label biomolecules both inside and outside cells. The basis of these approaches rely on the fact that the intensity profile generated from a point source of light is a known distribution, and is typically well approximated by a Gaussian [28], [29]. This allows the exact position of a molecule to be estimated by Gaussian fitting, with an uncertainty that depends on the number of photons collected [28], [30]. However, for accurate fitting, the intensity profile generated by each fluorophore must not overlap with other nearby fluorophores, which, in the case of bacteria, means imaging only a few molecules per cell. The breakthrough in super-resolution localization microscopy came about with the ability to image only a small subset of fluorophores at any one time by exploiting molecular photoswitching and photoactivation. Molecules are stochastically activated, imaged and localized over a movie with typically several thousand frames. The localizations from all frames can then be reconstructed into a super-resolved image (Fig.2D) [26], [31].

This can be achieved with organic fluorophores, using a buffer to induce photoblinking (dSTORM, [32]). However, this typically involves fixing and permeabilizing cells for labelling. Since our review focuses on live-cell methods, we will mainly discuss photoactivated localization microscopy, PALM [31], a method that relies on photoswitchable or photoactivatable variants of fluorescent proteins, such as mEos2 [33], Dendra2 [34] or PAmCherry [35]. These proteins can be photoactivated with near-UV light (∼400 nm), the intensity of which can be chosen to ensure that there are very few emitting molecules in a bacterial cell at any given time. Since these proteins are genetically encoded, this approach is well suited to live-cell microscopy. It is worth noting that dual approaches have been used to combine live-cell imaging followed by cell fixation and permeabilization on the slide and dSTORM imaging [36]. However, these techniques remain difficult to implement.

### 2.2 Preparation of fluorescent fusion strains

There is a large variety of photoactivatable fluorescent proteins (PAFPs), each with advantages and disadvantages in terms of their photophysical and biochemical properties. A primary concern for single-molecule imaging is minimizing interference from autofluorescence. In *E. coli* and many other bacteria, the autofluorescence is stronger towards the blue end of the spectrum, hence red PAFPs are common. Other factors which can influence the quality of super-resolution images are the brightness, photostability and blinking behavior of the PAFP. While the brightness of the fluorophore is important for localization precision, excellent photostability and minimal blinking characteristics are important factors for single-particle tracking experiments. Additional considerations include the oligomerization tendency of FPs, since this could cause undesired aggregation of target proteins. Most fluorescent proteins have been engineered to be monomeric, yet it has been shown that even among these variants, undesired aggregation may occur [37]. The folding speed, and the fraction of PAFPs which become fully mature are also important, especially for extracting copy numbers from PALM, since these properties determine the fraction of target-PAFP fusion proteins detectable in a cell [38]. For comprehensive comparisons between the properties of different PAFPs, we refer the reader to Refs. [37], [38], [39], [40].

There are several factors to consider when choosing labelling strategies. For example, the endogenous gene can be replaced with the fusion gene, or fusions can be expressed exogenously on plasmids. Replacing and inactivating endogenous copies of genes in *E. coli* can be performed with lambda red recombination [41], and can be moved between strains using P1 phage transduction [42]. Replacing the chromosomally encoded gene ensures that all copies of the target proteins are replaced by the fusions, which can make it simpler to test the functionality of the fusion protein. In general, it is critically important to check the functionality of any fusion protein, since even simple changes, such as the length of linker used, can alter its activity. Flexible linkers of 5–15 residues in length between the protein and the FP are most common, but longer linkers, and more rigid alpha-helical linkers can also be used [43]. In terms of the location of the FP group, C-terminal fusions are preferable since observation of FP fluorescence is clearly associated with fully translated tagged proteins; however, N-terminal as well as internal fusions are also viable options.

As an alternative to FPs, genetically encoded tags, such as HaloTag and SNAP-tag [44], [45], can be used to image in live bacteria [46]. These tags bind tightly and form covalent bonds with a membrane permeable ligand, which can be modified with organic fluorescent dyes; such dyes are typically brighter and much more photostable than FPs. To implement these labeling methods, live cells expressing the protein-tag fusion are incubated with a labelled ligand, and extensive washing removes any unreacted ligand, leaving only the ones that have reacted with the HaloTag or SNAP-tag.

### 2.3 Microscope design

Imaging single molecules inside cells requires specialized, sensitive microscopes. These typically feature high numerical-aperture objectives and electron multiplying CCD (EMCCD) or scientific CMOS (sCMOS) cameras which maximize the collected signal. Lasers are typically used as excitation sources, since their narrow frequency spectrum reduces unwanted background fluorescence, and makes it easier to precisely filter out excitation light. As super-resolution microscopy becomes more widely adopted, several commercial systems offering these features are becoming available. However, home-built set-ups still offer greater flexibility and can be optimized for specific experimental systems, for example, by selecting lasers and dichroic mirrors to match the fluorescent proteins used.

Here we describe a simple home-built total internal reflection fluorescence (TIRF) set-up for single-molecule localization microscopy (Fig.2A). TIRF microscopes reduce the unwanted signal caused by excitation of out-of-focus fluorescence, since the evanescent excitation extends only ∼150 nm into the sample [47]. For imaging more deeply into the cell, TIRF systems can be used at sub-critical angles giving a highly-inclined thin sheet of excitation light [48]. To record transmitted light images of cells (Fig.2B), an LED light source and condenser are positioned above the objective.

For PALM imaging, the microscope requires two lasers; one for photoactivation of PAFPs, and another for excitation. For photoactivation, a low power (1 mW) 405-nm laser is sufficient, since power densities up to 1 W/cm^2^ are typically used. A 100-mW 561-nm laser is used to excite photoactivatable red fluorescent proteins. Excitation laser power densities can be much higher (in the kW/cm^2^ range) to increase localization precision, although this comes with the cost of faster photobleaching and higher cell toxicity. Additional excitation lasers can be added for multicolor imaging, although since FPs have long emission tails, the additional excitation filters needed can reduce signal-to-noise ratios.

The lasers are first coupled into a single-mode fiber; at the fiber output, the excitation beam is collimated (using a 50-mm achromatic lens) and focused (using a 250-mm achromatic lens) in the back focal plane of the objective (100x oil-immersion objective, NA 1.4, focal length of 1.8 mm). To allow adjustment of the incident angle of the beam at the coverslip from TIRF to epifluorescence, the fiber output, collimation and focusing lenses are mounted on a translation stage controlling the position of the beam in the objective.

In ‘objective-type’ TIRF, the fluorescence emission is collected by the same objective used to introduce the excitation light. The excitation beam and emission signal are separated with a polychroic mirror and emission filter. A single tube lens (300 mm achromatic lens) focusses the emission signal onto the camera. The 300 mm tube lens and 1.8 mm focal length objective gives 167× magnification. A 512 by 512 pixel EMCCD camera is used to increase the signal-to-noise from imaging a single fluorophore (Fig.2C). An autofocus systems can be a useful addition to avoid drift while taking long PALM movies. An infrared LED can be used to minimize unwanted interference with the fluorophores under study. The infrared signal is delivered to and from the objective with its own long-pass dichroic mirror.

### 2.4 Sample preparation

A detailed protocol for growing *E. coli* cell cultures and preparing microscopy slides is given in Appendix A. One of the key experimental challenges for *in vivo* single-molecule imaging is minimizing unwanted background fluorescence. Much can be done in this regard during the preparation of samples, for example, glass coverslips should be carefully cleaned to remove fluorescent contaminants. One method to do this is by heating coverslips in an oven to 500 °C for one hour. Further, undefined growth media, such as lysogeny broth (LB), can contribute significantly to background fluorescence. For this reason, cells are typically grown in defined media; e.g., for *E. coli* one can use M9 minimal media, or rich defined media (EZRDM, Teknova) for faster growth. Similarly, low fluorescence agarose can be used to immobilize cells on the slide.

### 2.5 Generating super-resolved images of live cells

For localization microscopy, numerous algorithms [49] are available to accurately localize point sources from fluorophore images and reconstruct super-resolved images (Fig.2D). A popular approach is to first determine candidate positions for each fluorophore, followed by Gaussian fitting to extract precise localizations. This is done for each frame in the movie, and the super-resolved image is generated by collapsing all localizations onto a single image.

**Fig. 3:**
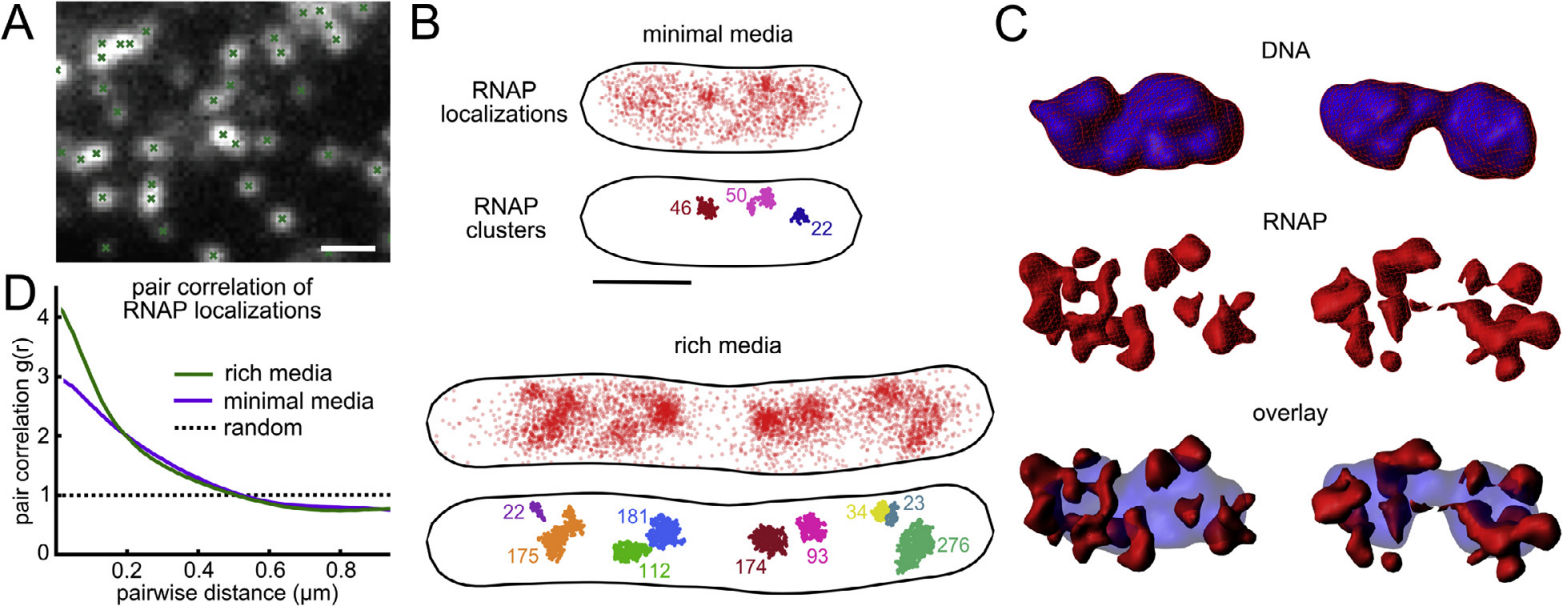
Generating and analyzing super-resolved images of live cells. A) An example field of view with a high density of photoactivated PAFPs. Localizations are identified with a crowded-field algorithm. B) Rapid-acquisition (15 s) PALM images RNAP in live *E. coli* analyzed with a crowded-field localization algorithm. Comparing slow (top) and fast (bottom) growth conditions, highlights increased clustering of RNAP in fast growth conditions. This can be quantified using a clustering algorithm. C) Super-resolved images of RNAP (red) and DNA (blue) imaged with 3D SIM. D) Pair correlation analysis of RNAP localizations in panel B. Panel A adapted from [51], panel B-D adapted from [65]. (For interpretation of the references to color in this figure legend, the reader is referred to the web version of this article.)

For live-cell microscopy, there are several additional factors to consider compared to imaging chemically fixed cells. For example, the fluorescence spot from fast-moving molecules may be motion-blurred, requiring localization by free elliptical Gaussian fitting. To counteract motion blurring, stroboscopic illumination can be used, with short (<5 ms) excitation laser pulses with a longer duration camera frame time [50]. Additionally, PALM data typically require thousands of frames (taking several minutes to acquire); this long experiment duration can limit live-cell imaging, since cellular features, such as the positions of genes, may move during this period. For faster acquisition, a higher photoactivation rate can be used, resulting in high-density images with overlapping fluorophore spots (Fig.3A). These can be analyzed with specialized crowded-field localization algorithms [51]; we applied this technique to generate snapshot images (acquired in ∼15 s) of RNAP localizations in live *E. coli* (Fig.3B).

SIM imaging is typically much faster than PALM imaging, requiring just tens of frames to reconstruct an image rather than thousands. SIM imaging is also well suited to multicolor imaging of RNAP together with the nucleoid, stained with an intercalating DNA dye. Fig.3C shows a surface rendering of 3D images of RNAP and the nucleoid acquired in ∼3 s, highlighting dense regions of RNAP. On the other hand, SIM imaging cannot achieve the same resolution as PALM; it is also harder to extract quantitative information from SIM images.

### 2.6 Single-particle tracking PALM

Tracking the movement of molecules in live cells is a powerful and direct means to observe the kinetics and location of protein activities. Combining single-particle tracking with the strategy of photoactivation central to PALM (sptPALM, [52]) allows many molecules to be tracked sequentially. As in typical PALM studies, single molecules are sparsely photoactivated and imaged for a number of frames. One of the key limitations of *in vivo* single-molecule techniques is photobleaching. Trajectories of single PAFPs are typically limited to only four or five frames (frame times vary between 1 and 100 ms, depending on the mobility of the protein being studied; for RNAP, we have used 15 ms) before photobleaching, which makes observing processes with slow kinetics more difficult. Compared to ordinary PALM, lower excitation intensities allow molecules to be tracked for a longer duration at the cost of decreased localization precision [21], [53].

### 2.7 *In vivo* perturbations

As genetic manipulation of cells becomes easier, ever more complex molecular biology assays can be performed *in vivo*. Overexpression of unlabelled versions of the protein under study, or partner proteins can be used to titrate certain interactions [54]. For example, the *in vivo* dissociation constant of protein complexes can be measured by comparing the mobility of each labelled subunit in unperturbed cells to the mobility after complex formation is prevented (e.g., by competition provided via overexpression of unlabelled interaction partners [55]). Even complex *in vitro* experiments can be recapitulated *in vivo*, such as a single-molecule chase assay where timed expression of unlabelled copies of a transcription factor was used to study the dissociation of fluorescent transcription factors from their operator site on DNA [24]. Tightly controlled inducible genes can be at positioned at specific chromosomal loci to test the effect on other processes [56], and CRISPR technology can be used to selectively silence or activate existing genes of interest [57], [58], or block other processes, such as DNA replication [59].

Small molecule inhibitors and antibiotics can also provide useful controls for live-cell experiments, and have the benefit of being easy to implement. For example, the antibiotic rifampicin blocks transcription by binding to RNAP and preventing elongation past the 3rd nucleotide, leaving an RNAP molecule stuck at the promoter site; however, rifampicin does not affect transcription by RNAPs already in transcription elongation, which proceed to complete transcription and dissociate from the DNA [27].

## 3 Quantitative imaging and data analysis

### 3.1 Analyzing spatial clustering

Localization microscopy images inherently lend themselves to quantification. Information about the spatial organization can be evaluated with clustering algorithms, such as DBSCAN [60], or more recently developed algorithms designed specifically for analyzing localization microscopy data [61], [62]. RNAP is known to increase its clustering as cell growth rate increases [63], as Fig.3B also demonstrates. While clustering typically requires defining thresholds which can alter the results, one can employ pair-correlation analysis, which offers an assumption-free method to assess the clustering of a sample [64] (Fig.3D). When calculating the pair correlation, it is necessary to normalize by the average density within the cell; corrections should also be made for the small size of the bacteria, since even at short radii, much of the region can fall outside the cell boundary [65].

### 3.2 Estimating copy numbers

With PALM imaging and tracking, each individual photoactivation events ideally represents a single molecule, which naturally allows counting protein copy numbers in single cells. To estimate copy numbers, all available PAFPs must be imaged. For highly expressed proteins with copy numbers over 10,000, this requires tens of thousands of frames of PALM acquisition. Over the course of the movie, photoactivation intensity must be controlled to ensure that there is at most a single active fluorescent molecule per cell.

Cells can be segmented based on the transmitted light image, and there is excellent software available for this [66], [67]. The total number of activated and imaged PAFPs can then be estimated by tracking the localizations falling within the segmented cell boundary. For RNAP, the mean copy number per cell measured in this way is ∼2700 for slow growth conditions [65], and ∼4600 for fast-growth conditions [22].

It is important to note that several sources of over-counting and under-counting can affect copy number estimates from PALM imaging. Photoactivatable proteins may blink after activation by transitioning from photoactive to dark states reversibly [68]. This effect, along with the transient passage of fluorescent molecules in and out of the area of illumination, can cause single molecules to be counted multiple times. Some blinking characteristics can be dependent on activation and excitation light, as well as buffer conditions, so it is important to conduct control experiments under exactly the same conditions to calibrate for these factors. Choosing PAFPs with minimal blinking can help to minimize overcounting [69], and some tracking algorithms can allow for a number of transient dark frames to account for any blinking or loss of localization.

On the other hand, sources of undercounting include the presence of unfolded and immature fluorescent proteins. It has been estimated that up to 50% of some PAFP variants do not fully mature [38], although this characterization was performed in *Xenopus* oocytes, and the maturation in bacteria may be very different. We have used PALM in *E. coli* to measure the copy numbers of DNA polymerase 1, which gave values ∼20% higher than those reported in the literature (∼480 compared to 400) [53].

### 3.3 Diffusion analysis

**Fig. 4:**
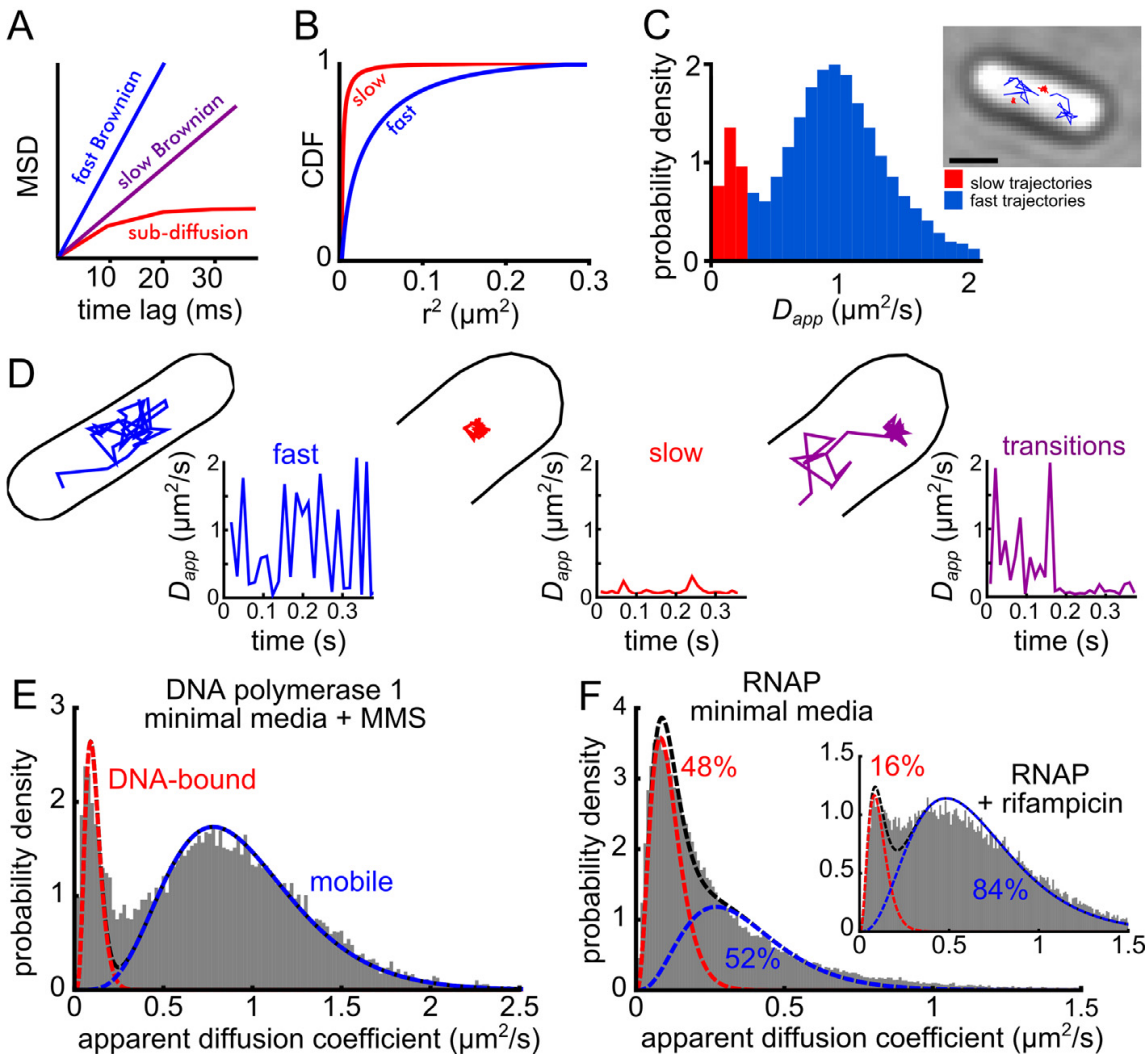
Analyzing single-particle tracking PALM data. A) Plotting the mean squared displacement of the sptPALM trajectories against time lag can provide information about the mobility of the labelled protein, and establish if motion is Brownian (where MSD increases linearly with increasing time lag) or sub-diffusive. B) Cumulative distribution of the squared displacements. This distribution can be fitted with Eqs. (2), (3), to extract information about the mobility of the proteins and the number of diffusive species. C) Distribution of apparent diffusion coefficients (*D_app_*) calculated for each single-molecule trajectory. A threshold can be used to sort individual trajectories based on their *D_app_* value, as shown in the example cell with slow trajectories colored red and fast trajectories colored blue (right). D) Examples of long trajectories (ten or more localizations) classified according to their *D_app_* transitions: a fast diffusing molecule, with a high average *D_app_* value over the whole trajectory (blue), a slow-moving molecule, with a low average *D_app_* value (red), and a molecule undergoing transition from fast (high *D_app_*) to slow (low *D_app_*) (purple). E) The *D_app_* distribution for DNA polymerase 1 treated with a DNA damaging agent to recruit molecules to DNA. The distribution shows two clearly resolvable peaks, which can be fitted with a two-species model (using Eq. (6)) to extract fractions of molecules in the low-mobility DNA-bound state, and the mobile state. F) The distribution of RNAP *D_app_* values can also be fitted with a two-species model. Treatment with rifampicin blocks transcription, causing a large drop in the fraction of DNA-bound RNAPs (inset). Panels A–C adapted from [73]. Panel D adapted from Ref. [55]. Panels E,F adapted from [65]. (For interpretation of the references to color in this figure legend, the reader is referred to the web version of this article.)

Trajectories generated from single-particle tracking experiments can be analyzed in several ways. Plotting the mean squared displacement (MSD) of many trajectories measured at the different time intervals, τ, can be used to determine if the diffusion is Brownian or sub-diffusive (due to confinement within 3D structures), and the slope of the plot can be used to determine the mobility. The MSD for trajectories measured in two-dimensions is given by:

(1) $$\mathrm{MSD}(\tau )=\frac{1}{N}\overset{N}{\underset{i=1}{\sum }}{\left[x_{i}(t_{0}\;+\;\tau )-x_{i}(t_{0})\right]}^{2}+{\left[y_{i}(t_{0}\;+\;\tau )-y_{i}(t_{0})\right]}^{2}$$

where *N* is the number of trajectories, and $x_{i}$ and $y_{i}$ are the coordinates of the trajectory. This analysis can be used to determine if the population is generally slow or fast moving [50], however, it becomes harder to interpret if multiple species with different diffusive behavior are present (Fig.4A). Alternatively, the distribution of the squared displacements, *r*^2^, for molecules taken at one particular time lag can be plotted as an empirical cumulative distribution function (CDF; Fig.4B). The resulting curve can be fitted to an analytical expression for the CDF for single or multiple diffusing species to extract estimates for the diffusion coefficients of these molecules and determine the fractions of molecules in different states [70], [71]:

(2) $$f(r^{2})=1-\exp\left(-\frac{r^{2}}{4\tau D}\right)$$

Additional species can be added in the same form.

(3) $$f\left(r^{2}\right)=A_{1}\left[1-\exp\left(-\frac{r^{2}}{4\tau D_{1}}\right)\right]+(1-A_{1})\left[1-\exp\left(-\frac{r^{2}}{4\tau D_{2}}\right)\right]$$

where $A_{1}$ is the fraction of the squared displacements from the diffusive species with diffusion coefficient $D_{1}$, with the remainder of the population being in diffusive state $D_{2}$.

Finally, an ‘apparent’ or ‘nominal’ diffusion coefficient $D_{\mathrm{app}}$, can be determined for each trajectory based on the single-step MSD (Fig.4C):

(4) $$D_{\mathrm{app}}=\frac{1}{4n\tau }\overset{n}{\underset{i=1}{\sum }}{\left[x_{i+1}-x_{i}\right]}^{2}+{\left[y_{i+1}-y_{i}\right]}^{2}$$

where $x_{i}$ and $y_{i}$ are the coordinates of the molecule at position i in the single-molecule trajectory, and n is the number of independent steps in the trajectory with time interval τ. The distribution of the $D_{\mathrm{app}}$ values, calculated from trajectories of length *n* can be fitted to an analytical expression [72]:

(5) $$f(D_{\mathrm{app}})=\frac{1}{(n-1)!}\cdot {\left(n/D\right)}^{n}\cdot {\left(D_{\mathrm{app}}\right)}^{n-1}\cdot \exp\left(-\frac{{\mathrm{nD}}_{\mathrm{app}}}{D}\right)$$

Since trajectories measured with single-particle tracking vary in length, trajectories shorter than n steps must be discarded, and longer trajectories truncated. For two species, the equation above becomes:

(6) $$f(D_{\mathrm{app}})=A_{1}\left[\frac{1}{(n-1)!}\cdot {\left(n/D_{1}\right)}^{n}\cdot {\left(D_{\mathrm{app}}\right)}^{n-1}\cdot \exp\left(-\frac{{\mathrm{nD}}_{\mathrm{app}}}{D_{1}}\right)\right]+(1-A_{1})\left[\frac{1}{(n-1)!}\cdot {\left(n/D_{2}\right)}^{n}\cdot {\left(D_{\mathrm{app}}\right)}^{n-1}\cdot \exp\left(-\frac{{\mathrm{nD}}_{\mathrm{app}}}{D_{2}}\right)\right]$$

Generating a $D_{\mathrm{app}}$ value for each trajectory allows the mobility information to remain linked to the spatial information for each molecule, thus helping the analysis of the location of different molecular species within in the cell, a treatment also amenable to color coding and intuitive visual inspection (Fig.4C) [73].

Molecules can sometimes be observed as they transition between diffusive states. These events can be distinguished by calculating a moving average $D_{\mathrm{app}}$ value over the course of long trajectories (for example, >10 steps) and identifying transitions across a threshold value (Fig.4D) [55]. However, since trajectories from sptPALM are typically short, care should be taken to make sure analyzing only a small subsection of trajectories does not introduce biases. Alternatively, a software package has been created to extract transition rates using Bayesian analysis of sptPALM trajectories of all lengths [74].

### 3.4 Simulating diffusion in cells

The apparent diffusion coefficients measured experimentally through particle tracking do not take into account confinement due to the small size of bacteria, and effects such as localization error and motion blurring. To address these issues and gain more detail into the underlying motion, several studies have used simulations of diffusion in cells to recapitulate experimental data [20], [50], [55], [65]. In our studies, we have simulated Brownian motion confined within a volume corresponding to the average size of cells imaged in experiments; e.g., for *E. coli*, we defined this as a cylindrical volume 2 µm long and 0.9 µm wide with hemispherical endcaps of a 0.9 µm radius. Each frame is split into sub-frames with Gaussian-distributed displacements in each sub-frame, and each molecular trajectory given a random starting time to mimic stochastic photoactivation. The trajectory is then simulated with a duration sampled from an exponential distribution with a mean time equal to our experimentally determined photobleaching lifetime (typically 4–6 frames long). The sub-frame distributions can then be averaged to give a position for each frame, and a localization error added. The list of simulated localizations along with their corresponding frame number can then be analyzed using the same tracking algorithm with the same settings used for the experimental data.

## 4 Imaging transcription

### 4.1 Using sptPALM to determine the fraction of RNAP transcribing genes

In *E. coli*, chromosomal loci move sub-diffusively, with an MSD of ∼10^−2^ µm^2^ measured at 1 s time intervals [75]. On the other hand, individual proteins not interacting with DNA can have a mobility several orders of magnitude higher [76]. For example, estimates of the diffusion coefficients of freely diffusing unconjugated fluorescent proteins in *E. coli* range from 7 m^2^/s [77] to 10 µm^2^/s [78]. For RNAP, this difference in mobility between DNA-bound molecules and mobile molecules (the latter representing diffusing and transiently binding RNAPs) can be exploited to distinguish transcribing RNAPs from the rest of the population.

To establish the apparent diffusion of the DNA-bound RNAP species, control proteins can be used. As a PALM standard for a DNA-bound protein, one can use a PAFP fusion to DNA polymerase I (Pol1), which shows clearly distinct populations for molecules specifically bound to DNA and those searching the chromosome for substrates (see Ref [53], and Fig.4E of our paper). Fitting this $D_{\mathrm{app}}$ distribution using Eq. (6) allow us to establish the *D* value of specifically bound molecules. The apparent motion of bound molecules is mainly due to the localization uncertainty in each measurement, $\sigma_{\mathrm{loc}}$, which manifests itself as a positive offset in the *D* value of $\frac{\sigma_{\mathrm{loc}}^{2}}{\tau }$ [79]. This corresponds to ∼0.1 µm^2^/s for $\sigma_{\mathrm{loc}}=40\text{nm}$.

Fitting the RNAP $D_{\mathrm{app}}$ distribution using a two-species model that includes the DNA-bound population and a second *D* species linked to the population of mobile RNAP molecules showed that ∼48% of RNAPs were bound and ∼52% were mobile in slow cell growth conditions (Fig.4F) [65]. This result agrees with previous estimates from fluorescence recovery after photobleaching (FRAP) studies on fluorescently labelled RNAP in *E. coli* [80]; the FRAP work (which was averaged over many cells) showed that, ∼53% of the RNAP molecules were mobile on the 3 s time-scale, and the remaining 47% were immobile even on the 30 s time-scale. The fraction of RNAPs which are transcribing is sensitive to growth rate, and the fraction of bound RNAPs increased to 63% in fast growth conditions [65]. Blocking transcription with rifampicin (see Section 2.7) leads to a clear decrease (from 48% to 16%) in the fraction of DNA-bound RNAPs (Fig.4F, inset).

### 4.2 Spatial organization of transcription and the nucleoid

Transcription plays a central role in maintaining both global and local chromosome organization. Growth conditions influence both transcriptional activity and nucleoid structure. During slow growth, the nucleoid lacks observable structure; however, during fast growth, the nucleoid displays dramatic variation in local DNA density [81]. Over this range of growth rates, the overall rate of synthesis of ribosomal RNA increases ∼40-fold, whereas most other genes are down-regulated [82].

Direct imaging of labelled RNAP in cells has been used to study these growth-dependent effects. Initial work with conventional fluorescence microscopy in fixed cells demonstrated that changes in the level of expression is reflected in large changes in the spatial distribution of RNAP: at slow growth conditions, RNAP appears to be fairly homogeneously distributed over the diffuse nucleoid, whereas at fast growth conditions, dense clusters of RNAPs emerge [63], [69]. These dense clusters have been likened to “transcription factories” in eukaryotic cells, where a single site contains multiple RNAPs active on different genes [83]. Using rapidly acquired PALM snapshots (see Section 2.5, Fig.3B), these clusters can be visualized in live cells. Quantifying the size of the clusters with both a density based clustering algorithm, and pair correlation (see Section 3.1, Fig.3B,D), demonstrated that the numbers of RNAPs in each cluster are much larger at faster growth rates than slow growth rates.

**Fig. 5:**
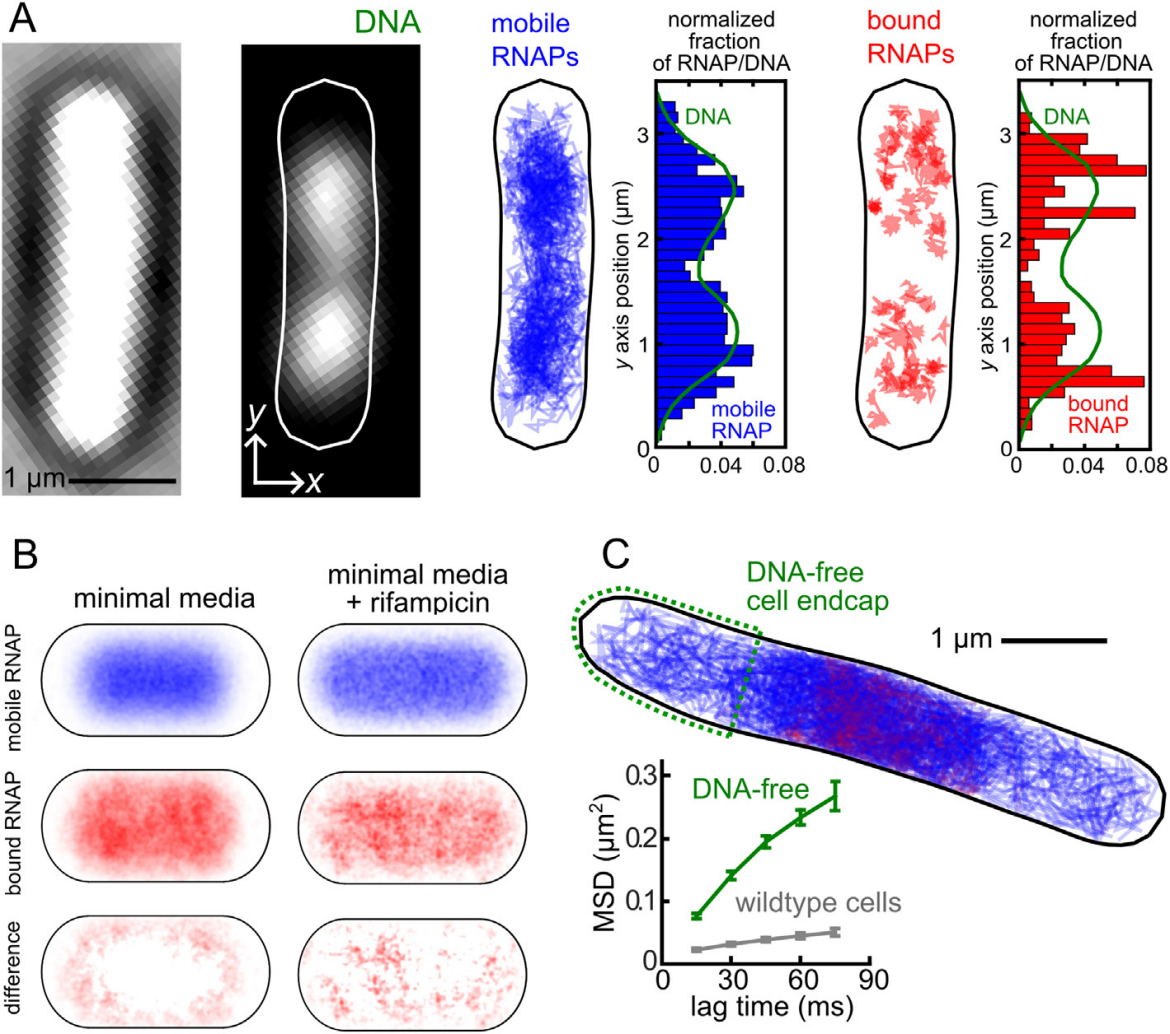
Spatial organization of transcription and non-specific DNA interactions. A) Transmission image of an example cell, and an image of DNA stained with an intercalating fluorescent dye. The distribution of sorted mobile RNAP trajectories (blue lines/bars) closely matched the distribution of DNA (green line). The distribution of bound RNAPs in the same example cell shows a more clustered distribution which does not closely follow the distribution of DNA. B) Spatial distribution of sorted RNAP trajectories averaged over ∼200 cells between 1.6 and 2.5 µm long. Transcribing RNAPs show a bias towards the periphery of the nucleoid region, which is lost after blocking elongating RNAPs with rifampicin. C) Example ‘minimal-DNA’ cell (top); temperature-sensitive DnaC mutant cells are grown at a non-permissive temperature to give long cells with a single centrally located chromosome. Tracking RNAPs only in the DNA-free cell endcaps (green dashed region) allows the free 3D diffusion to be determined. The mean squared displacement (bottom) shows that the diffusion of RNAP in DNA-free cell endcaps (green line) is much faster than the average diffusion of RNAP molecules in normal unperturbed cells (grey line). Panels A–C adapted from [65]. (For interpretation of the references to color in this figure legend, the reader is referred to the web version of this article.)

Using sptPALM to sort transcribing RNAPs based on their mobility has also revealed that active transcription reorganizes the positions of genes. In this analysis, an apparent diffusion coefficient is calculated for each RNAP trajectory (see Section 3.3), and a threshold is introduced to separate more mobile RNAPs from slower moving molecules, which are likely to be transcribing genes on DNA. The spatial distribution of these sorted trajectories gives a valuable insight into where transcription is taking place (Fig.5A). The average spatial distribution over hundreds of cells can be plotted by segmenting the cells based on the transmission image and determining the positions of trajectories relative to the cell membrane and cell midline (Fig.5B). These plots show that transcribing RNAPs is biased towards the periphery of the nucleoid region, but this organization is lost when active transcription is blocked with rifampicin. SIM imaging (Fig.3D) confirmed that the densest regions of RNAP (corresponding to the most highly transcribed genes) were located at the edge of the nucleoid, where the density of DNA is low.

### 4.3 The target search process

*In vivo* tracking has proved to be an excellent tool for studying how the proteins involved in transcription locate their target in cells. Transcription factors are responsible for controlling much of gene expression in cells, and extensive work has been performed in live cells to study both the specific and non-specific DNA interactions of the transcription factor lac repressor (LacI), a protein responsible for regulating lactose metabolism [19], [23]. By using a mutant of LacI with its DNA binding domain removed, the free 3D diffusion of LacI was measured. The fraction of time which the protein spends interacting non-specifically with DNA, x, can be estimated using the equation [19]:

(7) $$D_{\mathrm{searching}}={\mathrm{xD}}_{\mathrm{bound}}+(1-x)D_{\mathrm{free}}$$

where $D_{\mathrm{free}}$ is the free 3D diffusion coefficient (measured with a non-DNA binding mutant), $D_{\mathrm{bound}}$ is the diffusion coefficient of DNA-bound molecules, and $D_{\mathrm{searching}}$ is the diffusion coefficient of mobile molecules searching through a combination of transient DNA interaction and 3D diffusion. Using this equation, it was shown that LacI spends ∼90% of its search time bound non-specifically to DNA.

Comparing the spatial distribution of mobile RNAP molecules with that of DNA, shows the mobile RNAPs are very highly associated with the nucleoid (Fig.5A). This suggests a high level of transient interactions with DNA, as well as 3D diffusion between strands of DNA during the search process, and suggests that RNAP can access even the densest regions of the nucleoid. Similar approaches to earlier studies of LacI have been employed to study the non-specific DNA binding of RNAP; however, since RNAP is a large multisubunit protein with many interactions with DNA, it is not straight-forward to create RNAP mutants which show no interactions with DNA. As an alternative, one study has used sptPALM with very short exposure times (2 ms/frame) to directly image freely diffusing RNAP [20]; this study provided quantitative estimates of the fraction of RNAP in different states, showing that RNAP spends ∼70% of its search process interacting non-specifically with DNA.

We adopted an alternative approach to measure the free diffusion of RNAP, by creating a ‘minimal-DNA’ strain carrying a temperature-sensitive DnaC mutation. At non-permissive temperatures, DnaC(ts) cells are unable to initiate DNA replication but keep elongating, yielding long cells containing a single chromosome and long DNA-free endcaps [84]. Tracking RNAP molecules located only in the DNA-free cell ends allowed us to determine the *D* of free RNAP (Fig.5C). Using Eq. (7), we showed that RNAP spends ∼85% of its search process interacting non-specifically with DNA. Taken together, these two estimates of RNAP non-specific binding provides strong evidence that, similar to LacI, a large majority of the promoter search process is spent by the RNAP transiently interacting with DNA.

## 5 What does the future hold for *in vivo* single-molecule transcription studies?

The powerful combination of PALM imaging with single-particle tracking has already provided a wealth of information on the copy number, mobility, sub-cellular distribution, and spatial organization of RNAP. There are several extensions, however, that will further increase the information content from this super-resolution approach, and help apply the basic methods to more complex systems and settings. These extensions broadly fall into advanced high-resolution microscopies; correlative measurements with other transcription components; longer timescales for kinetics; advanced data-analysis routines; and applications to eukaryotic transcription.

Advanced PALM methods will provide additional contrast on the RNAP location relative to cellular structures (cell membrane, nucleoid). One such method is 3D PALM imaging and tracking, which can rely on astigmatism, bifocal optics, or PSF-engineering methods (see Ref [26] for a review). This has been applied in bacteria [85]. PALM studies in bacteria will also benefit from increased use of microfluidics [86], which will increase the measurement throughput, and provide controllable means to maintain or change the physiology of cell populations under study. Two-color PALM measurements will also help visualize the relative spatial arrangement of interacting proteins, as well as the location of RNAPs relative to the nucleoid or RNA molecules at high-resolution. Such measurements are usually complicated due to suboptimal fluorophore combinations, but improvements in PAFPs and in microscopy will enable such studies; pairs such as PAGFP – PATagRFP [87] and rsKame – PAmCherry [88] provide viable alternatives for two-color PALM.

New insight of transcription mechanisms *in vivo* will no doubt be obtained by labeling and tracking different components of transcription machinery. This effort may involve labeling of sigma factors, transcription factors, and nascent RNA (e.g., using MS2- and MS2-like RNA-visualization strategies; [89]). Depending on the copy numbers, one may be able to choose between PAFPs and proteins that do not require photoactivation, thus increasing the palette of available FPs and fluorophores. Further insight will be gained by relating the position of transcription proteins to *specific* genomic sites, which can be labelled using small FROS systems or the ParB-*parS* system; use of smaller tags will be preferable [90], since it is less likely that the DNA probe will affect the location of the labelled DNA locus.

The ability to extend tracking to timescales comparable to those for the transcription of an entire gene (∼1 min) will be transformational, since it will permit monitoring entire rounds of target search and transcription and relating them to the physiological state of bacteria, as well as to the location and sequence of genes involved. Use of stroboscopy, time-lapsed acquisition, and use long exposures to visualize bound molecules can extend the current timescale from ∼100 ms to ∼10 s. Further improvement of FPs (e.g., TagRFP [91]; mScarlet [92]) and PATagRFP [87], which are much more photostable than PAmCherry) should extend tracks further.

Moving from FPs to organic fluorophores will substantially increase the photon count and photostability of the fluorophores, thus improving localization precision and increasing track length by orders of magnitude. This can be achieved via protein labeling using SNAP or Halo-tags (see Section 2.2), or fluorescent unnatural amino-acids (recently introduced to nascent proteins during *in vivo* translation; [93]). Use of electroporation can also introduce proteins labelled with organic fluorophores [94], [95], as well as labelled DNA fragments that can act as transcription substrates. Apart from the extended timescale of observation, these developments can lead to lower powers used during PALM acquisition, which is bound to reduce potential effects of light on bacterial physiology.

Our ability to extract information from localization data and multi-color images will be further enhanced by powerful advanced data analysis methods. For example, the presence of multiple, interconverting diffusive states (corresponding to different complexes of RNAP, which may also have a different tendency for non-specific DNA binding) may be detectable using Hidden Markov Modeling (HMM) methods that have been developed and applied to RNA-interacting proteins in bacteria [74]. Data analysis methods can also be interfaced with closed-loop feedback control to adjust the photoactivation rate and ensure the low density required for PALM acquisition [68], [85].

Studying bacterial transcription with these methods has also provided a spring-board for more technically challenging studies of transcription in eukaryotic cells. To counteract the decreasing fluorescence signal at larger depths from the coverslip, light sheet microscopes have been used to study transcription factor dynamics and RNA polymerase II (PolII), in live mammalian cells [71], [96], [97]. Other studies have used PALM and pair correlation analysis to study PolII clustering dynamics in mammalian cells [98].

## Appendix A

1) Preparing cell cultures
  1.1) Streak an *E. coli* strain with a PAFP-target fusion gene from a frozen glycerol stock on a Luria Broth (LB) agarose plate with the appropriate antibiotics and incubate at 37 °C overnight.
  1.2) Inoculate a 5 ml LB liquid culture from a single cell colony and grow at 37 °C shaking at 220 rpm for 3 h.
  1.3) Dilute the culture into 5 ml of low-fluorescence growth media (e.g. M9 medium or rich defined media) and incubate at 37 °C shaking at 220 rpm overnight.
  1.4) The next day, measure the optical density (OD_600_) using a spectrophotometer and dilute the culture in 5 ml fresh growth medium. Grow for at least 2 generations at 37 °C shaking at 220 rpm to early exponential phase (∼OD 0.1 for minimal media or OD 0.3 for rich media).
  1.5) Concentrate 1.5 ml of cells in a microcentrifuge tube by centrifugation at 2000*g* for 3 min. Remove the supernatant and resuspend the cell pellet in 10 µl of the residual medium.
2) Microscope slide preparation
  2.1) Prepare a 1% low-fluorescence agarose solution in the same growth media used during cell culture.
  2.2) Melt the agarose and pipette 500 µl onto the center of a microscope slide.
  2.3) Before the agarose has solidified, flatten the top surface by placing a coverslip on top. Note that coverslips should be carefully clean before use, for example by heating in an oven at 500 °C for one hour. Coverslips cleaned in this way can be stored for weeks in aluminum foil.
  2.4) Remove the coverslip from the pad and add 1 µl of the concentrated cell suspension onto the agarose pad. Immobilize the cells by re-covering the pad with the coverslip.
